# Experimental synthesis of partially coherent beam with controllable twist phase and measuring its orbital angular momentum

**DOI:** 10.1515/nanoph-2021-0432

**Published:** 2021-09-29

**Authors:** Haiyun Wang, Xiaofeng Peng, Hao Zhang, Lin Liu, Yahong Chen, Fei Wang, Yangjian Cai

**Affiliations:** School of Physical Science and Technology, Soochow University, Suzhou 215006, China; School of Physics and Electronics, Shandong Provincial Engineering and Technical Center of Light Manipulations & Shandong Provincial Key Laboratory of Optics and Photonic Devices, Shandong Normal University, Jinan 250014, China

**Keywords:** optical coherence, orbital angular momentum, partially coherent beam, twist phase

## Abstract

Twist phase is a nontrivial second-order phase that only exists in a partially coherent beam. Such twist phase endows the partially coherent beam with orbital angular momentum (OAM) and has unique applications such as in super-resolution imaging. However, the manipulation and the detection of the twist phase are still far from easy tasks in experiment. In this work, we present a flexible approach to generate a famous class of twisted Gaussian Schell-model (TGSM) beam with controllable twist phase by the superposition of the complex field realizations using a single phase-only spatial light modulator. The precise control of the amplitude and phase of the field realizations allows one to manipulate the strength of the twist phase easily. In addition, we show that the twist factor, a key factor that determines the strength of twist phase and the amount of OAM, can be measured by extracting the real part of the complex degree of coherence of the TGSM beam. The experiment is carried out with the help of the generalized Hanbury Brown and Twiss experiment as the generated TGSM beam obeys Gaussian statistics. The flexible control and detection of the twist phase are expected to find applications in coherence and OAM-based ghost imaging.

## 1 Introduction

Over the past three decades, light beams carrying orbital angular momentum (OAM) have been widely studied owing to their unique properties and diverse applications in optical tweezers, optical communications, nonlinear optics, and so on [1, 2]. Perhaps, the most known light beam carrying OAM is the vortex beam [3, 4]. Such beam possesses helical phase front i.e., exp(ilφ) , where l is the topological charge and φ is the azimuthal angle in polar coordinate [5]. In 1992, Allen and coauthors found that the vortex beam has a well-defined OAM, equivalent to lℏ per photon ( l is an integer number), where ℏ is the reduced Plank constant. This general result opens a new chapter in modern optics, i.e., singular optics [6]. The vortex beams and optical vortices can be generated and manipulated by various methods including the in-cavity and out-cavity approaches with the use of macroscopic and microstructural elements [3, 7], [8], [9]. Meanwhile, many approaches for the determination of the topological charge l have been proposed, such as with the slit interference [10], [11], [12], prescribed aperture diffraction [13], special diffraction gratings [14], coordinate transformation [15], mode conversion [16], and astigmatic transformation [17]. The generation and measurement of the vortex beam endowed with partial coherence have also been studied extensively [18], [19], [20], [21]. Besides the vortex phase, astigmatic phase is another phase that could induce OAM [22, 23]. An elliptical Gaussian beam passing through a cylindrical lens could generate such astigmatic phase. The advantage of astigmatic phase is that it could produce very high OAM, e.g., up to 10,000ℏ per photon.

Twist phase is another nontrivial phase that could induce the light beams carrying the OAM [24, 25]. Different from the vortex and astigmatic phases, the twist phase is a *second-order* phase that depends on two spatial points and cannot be separated with respect to two positions. Thus, the twist phase exists only in a partially coherent light and is encoded within the second-order coherence function. Compared with the fully coherent light, the partially coherent light has found advantages in many applications [26], [27], [28], [29]. The expression for the twist phase is $\exp\left[-i\mu_{0}\left(x_{1}y_{2}-x_{2}y_{1}\right)\right]$ , where $\mu_{0}$ is the twist factor, related to the amount of OAM carried by each photon and $\left(x_{1},y_{1}\right),\left(x_{2},y_{2}\right)$ are two arbitrary position vectors in the beam transverse plane. The twist factor is bounded by $\left|\mu_{0}\right|\leq \delta_{0}^{-2}$ , where $\delta_{0}$ is the transverse spatial coherence width of the light beam. We can find when the beam becomes fully coherent, i.e., $\delta_{0}$ tends to infinity, $\mu_{0}=0$ , and the twist phase disappears. Since Simon et al. introduced the twist phase in a Gaussian Schell-model beam, which is named the twisted Gaussian Schell-model (TGSM) beam, the propagation of the TGSM beam in paraxial optical systems, dispersive media, and uniaxial crystal has been well explored theoretically [30], [31], [32], [33], [34]. The OAM flux density and its interaction with vortex phase were also studied in detail [35]. Recently, the studies of the conditions for embedding the twist phase in other kinds of partially coherent beams and devising new kind of twisted beams have paid considerable attention [36], [37], [38], [39], [40]. The TGSM beam has found potential applications in super-resolution imaging, optical trapping, free-space optical communications, and beam self reconstruction enhancement [41], [42], [43], [44]. However, the experimental generation of the TGSM beam with controllable twist factor is far from an easy task. To the best of our knowledge, there are only a few reports to generate the TGSM beam experimentally. One way is to a transform anisotropic GSM beam to the TGSM beam by using six or three cylindrical lenses system [45, 46]. However, this approach is difficult to modulate the twist factor. Another method proposed recently is the incoherent superposition of the continuous coherent modes [47, 48]. In that method, the generated twisted beam does not obey Gaussian statistics as the modes are not randomly fluctuating. Thus, it is restricted in particular applications where the Gaussian statistics is required, e.g., in ghost imaging [49, 50]. In addition, the experimental measurement of the twist factor of the TGSM beam is still a tricky challenge as the TGSM beam has the very weak spatial coherence and therefore the traditional interferometric methods cannot be used. As far as we know, there is no report on the experimental measurement of the twist phase.

In this work, we introduce an efficient way to generate the TGSM beam with controllable twist phase with the aid of a single phase-only spatial light modulator (SLM). The methodology is based on the superposition of the random modes generated by the stochastic complex transmittance screens [51]. Thus, the generated TGSM beam obeys Gaussian statistics. Moreover, we show that the twist factor can be quantitatively measured from extracting the real part of the two-point complex degree of coherence (DOC) of the twisted source. A proof-of-principle experiment is carried out to determine the twist phase of the generated TGSM beam with the help of the generalized Hanbury Brown and Twiss effect [52]. Our results open a new avenue for manipulating the second-order phase and OAM of the partially coherent light and may find novel applications in optical trapping, imaging, and optical communications.

## 2 Theory

The second-order statistical properties of a TGSM beam, propagating along *z*-axis, is characterized by a two-point cross-spectral density (CSD) function in space-frequency domain [24]

(1) $$W\left(r_{1},r_{2}\right)=〈E^{\text{*}}\left(r_{1}\right)E\left(r_{2}\right)〉=\exp\left[-\frac{1}{4\sigma_{0}^{2}}\left(r_{1}^{2}+r_{2}^{2}\right)\right]\mu \left(r_{1},r_{2}\right)\text{,}$$

with

(2) $$\mu \left(r_{1},r_{2}\right)=\exp\left[-\frac{1}{2\delta_{0}^{2}}{\left(r_{1}-r_{2}\right)}^{2}\right]\exp\left[-i\mu_{0}{\left(r_{1}\times r_{2}\right)}_{⊥}\right]\text{.}$$

Above $\mu \left(r_{1},r_{2}\right)$ is the complex DOC function, $r_{1}=\left(x_{1},y_{1}\right)$ and $r_{2}=\left(x_{2},y_{2}\right)$ are two position vectors in the source plane ( z=0 ), E(r) denotes the electric field realization, the asterisk and the angular brackets stand for the complex conjugate and ensemble average over the source fluctuations, respectively. $\sigma_{0}$ and $\delta_{0}$ are the beam width and the spatial coherence width, respectively. The subscript ⊥ in Eq. (2) denotes the component of cross-product orthogonal to the propagation axis. $\mu_{0}$ is a twist factor, a measure of the strength of the twist phase. The magnitude of $\mu_{0}$ is bounded by the inequality $\left|\mu_{0}\right|\leq \delta_{0}^{-2}$ , which ensures the nonnegative definiteness of the CSD function. When **r** = **r**_1_ = **r**_2_, the CSD function reduces to the average intensity of the TGSM beam, i.e., S(r)=W(r,r)=〈I(r)〉 .

In order to synthesize the TGSM source experimentally, the DOC function can be written as the following alternative integral form

(3) $$\mu \left(r_{1},r_{2}\right)=\exp\left[-\alpha \left(r_{1}^{2}+r_{2}^{2}\right)\mu_{0}^{2}/2\right]\int \int p\left(v\right)H^{*}\left(r_{1},v\right)H\left(r_{2},v\right)d^{2}v\text{.}$$

The function p(v) and H(r,v) take the form

(4) $$p\left(v\right)=\alpha \exp\left(-\alpha v^{2}\right)/\pi \text{,}$$

(5) $$H\left(r,v\right)=\exp\left[\alpha \mu_{0}\left(xv_{y}-yv_{x}\right)\right]\exp\left[-i\left(xv_{x}+yv_{y}\right)\right]\text{.}$$

The parameters α , $\mu_{0}$ , and $\delta_{0}$ satisfy the relation: ${\left(2\delta_{0}^{2}\right)}^{-1}=\alpha \mu_{0}^{2}/4+{\left(4\alpha \right)}^{-1}$ . To produce the random fields whose correlation function have the complex DOC shown in Eq. (3), we let *H* be a realization of an optical field parameterized by random vector **v** = (*v*_*x*_, *v*_*y*_), and *p*(**v**) interprets as the joint probability function of **v**. Now, let us consider a form of stochastic optical field generated by a deterministic field *τ*(**r**) passing through a complex random screen *T*(**r**), given by E(r)=τ(r)T(r) . Taking the second-order statistic of the stochastic field, one obtains the CSD function.

(6) $$W\left(r_{1},r_{2}\right)=\tau^{*}\left(r_{1}\right)\tau \left(r_{2}\right)〈T^{*}\left(r_{1}\right)T\left(r_{2}\right)〉\text{.}$$

**Figure 1:**
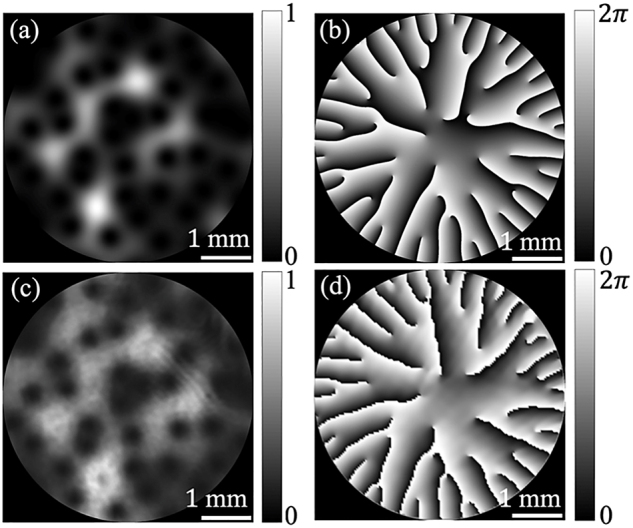
Theoretical results of the (a) intensity distribution and (b) phase distribution of one realization (instantaneous electric field). (c) and (d), the corresponding experimental results of the intensity distribution and phase distribution.

Compared Eq. (6) to Eqs. (1) and (3), one could establish the bridge between the complex DOC and the complex random screens, which is

(7) $$〈T^{*}\left(r_{1}\right)T\left(r_{2}\right)〉=\int \int p\left(v\right)H^{*}\left(r_{1},v\right)H\left(r_{2},v\right)d^{2}v\text{,}$$

and $\tau \left(r\right)=\exp\left\{-\left[{\left(4\sigma_{0}^{2}\right)}^{-1}+\alpha \mu_{0}^{2}/2\right]r^{2}\right\}\text{.}$ Following from the analysis in Ref. [51], the complex random screen T(r) can be evaluated from the following integral by changing the variable v=2πf , i.e.,

(8) $$T\left(r\right)=\sqrt{2\pi \alpha }{\int \int R\left(f\right)\left[2\pi^{2}p\left(2\pi f\right)\right]}^{1/2}H\left(r,2\pi f\right)d^{2}f\text{,}$$

where *R*(**f**) is the circular complex Gaussian random numbers with zero mean and unit variance. Eq. (8) can be evaluated numerically by means of the fast Fourier transform (FFT) algorithm. The procedure for the computing the single realization, say *T*_*n*_(**r**) (*n* = 1, 2, 3, …) of the stochastic field is as follows: The first exponential function in *H* function is evaluated at a desired location (*x*_*i*_, *y*_*j*_), then performing the two-dimensional FFT of Eq. (8). The result of the FFT at the location (*x*_*i*_, *y*_*j*_) is the true value of the screen *T*_*n*_(**r**). The above procedure is repeated for all (*x*_*i*_, *y*_*j*_) in the transverse plane. Finally, the obtained screen *T*_*n*_(**r**) is multiplied by a Gaussian function *τ*(**r**). Figure 1(a) and (b) show the simulation results of the distribution of the intensity and the phase of one realization of random electric field *E*(**r**). The parameters are chosen to be *σ*_0_ = 1.6 mm, *δ*_0_ = 0.4 mm, and *μ*_0_ = 6.25 mm^−2^. Obviously, the amplitude and phase fluctuate randomly in space.

In practical circumstance, one could express the ensemble average as the summation of large number of random electric fields, to be a good approximation, if the random process is stationary, i.e.,

(9) $$〈T^{*}\left(r_{1}\right)T\left(r_{2}\right)〉=\frac{1}{N}\sum_{n=1}^{N}T_{n}^{*}\left(r_{1}\right)T_{n}\left(r_{2}\right)\text{,}$$

where *N* is the number of realizations. Eqs. (7)–(9) provides an efficient way to experimentally synthesize the TGSM beams based on incoherent superposition of the random fluctuating fields. The modulation of the amplitude and phase of each realization can be realized with the help of particular optical devices, such as a spatial light modulator (SLM) or a digital mirror device (DMD). It is worth to note that one can conveniently control the strength of twist phase, i.e., twist factor, in the process of generating the random realizations *T*_*n*_(**r**) since the twist factor is contained in the kernel function *H*(**r**, **v**) shown in Eq. (6).

## 3 Experiment

### 3.1 Generation of a TGSM beam via random mode superposition

**Figure 2:**
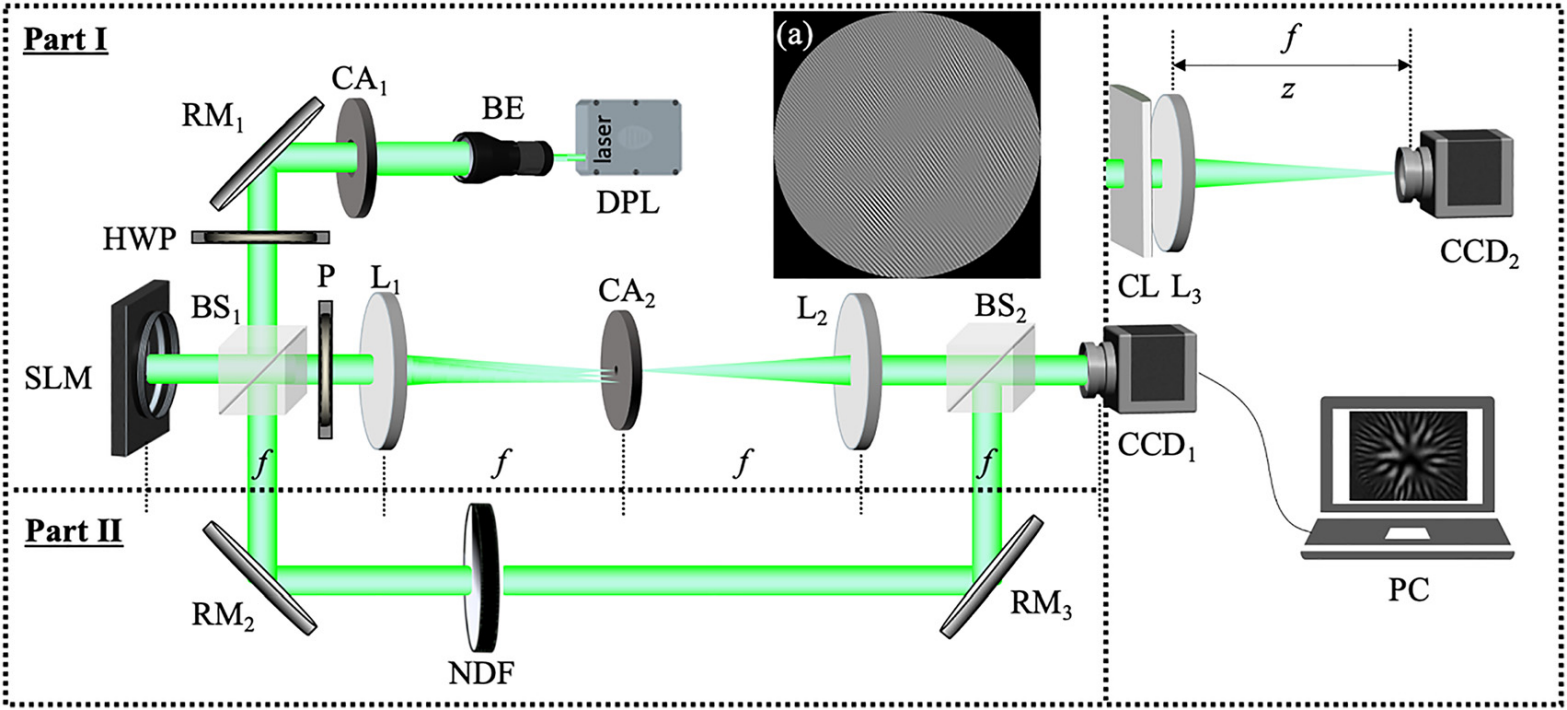
Experimental setup for synthesizing and measuring the TGSM beam with controllable twist phase. DPL, diode-pumped solid-state laser; BE, beam expander; CA_1_, CA_2_, circular apertures; RM_1_, RM_2_, RM_3_, reflect mirrors; HWP, half-wave plate; BS_1_, BS_2_, beam splitters; SLM, spatial light modulator; P, linear polarizer; L_1_, L_2_, L_3_, thin lenses; CL, cylindrical lens; NDF, neutral-density filter; PC, personal computers.

Part I of Figure 2 shows our experimental setup for generating the TGSM beam. A linearly polarized beam (*λ* = 532 nm) emitting from a diode-pumped solid state (DPSS) laser is expanded by a beam expander (BE) and reflected by a reflective mirror (RM_1_). We note here that the DPSS laser used here is a single longitudinal mode and TEM_00_ transverse mode laser (Cobolt Samba 532 nm laser). The beam then goes towards a beam splitter (BS). The transmitted portion entering part II is used as a reference wave to measure the twist factor of the generated TGSM beam, which we will discuss in the next subsection. The reflected portion impinges on a phase-only spatial light modulator (SLM, Pluto-VIS, Holoeye) on which a computer-generated hologram (CGH) is loaded to modulate the amplitude and phase of the incident beam. Although the phase-only SLM can modulate only the phase of the incident light, several methods have been proposed to simultaneously encode the amplitude and the phase information on a phase-only CGH. Here, we adopt the method for synthesizing the CGH of type 3 described in Ref. [53]. The basic idea is as follows: we first write the phase-only CGH as the form h(x,y)=exp[iψ(A,ϕ)] , where ψ is the function of the prescribed amplitude A and phase ϕ . Note that A and ϕ are the spatially dependent. The h(x,y) function is then expanded in terms of Fourier series and is associated with the phase modulation ψ(A,ϕ)=f(A)sin(ϕ) with f(A) being an unknown function. Finally, the function f(A) is solved by the equation $J_{1}\left[f\left(A\right)\right]=0.582A$ . The inset (a) in Figure 2 illustrates the typical CGH associated with the blazed grating to generate the single realization of the field.

The modulated light reflects from the SLM and passes through the BS again, entering a 4*f* optical system consisting of lenses L_1_ and L_2_. The use of the 4*f* system is to filter out the unwanted diffraction order and background noise with the help of the CA_2_ located in the rear focal plane of L_1_, and to image the modulated beam with unit magnification. The imaging plane is regarded as the source plane of the generated TGSM beam. The intensity and phase distributions of an instantaneous electric field measured from experiment are shown in Figure 1(c) and (d), corresponding to the theoretical results shown in Figure 1(a) and (b). One can see that the generated instantaneous field agrees reasonably well with the prescribed one. Since the TGSM beam is the incoherent superposition of a large number of realizations (randomly fluctuating electric fields), *N* = 5000 CGHs encoded with the random complex fields is prepared in advance and is stored in computer memory. The SLM operates in such a manner that at each time step, the chronologically earliest CGH is removed from the SLM’s screen and replaced by a new CGH. The SLM’s screen plays 5000 CGHs in cycle with each CGH being equal displaying time; about 18 ms. The CCD captures the intensity distributions of all realizations. The average intensity distribution of the TGSM beam can be obtained by averaging over the intensity of all realizations. In the experiment, we generate two TGSM beams with twist factors $\mu_{0}=6.25{\text{mm}}^{-2}$ and $\mu_{0}=-6.25{\text{mm}}^{-2}$ . This can be done by preparing two sets of CGHs, one for $6.25{\text{mm}}^{-2}$ and the other for $-6.25{\text{mm}}^{-2}$ .

**Figure 3:**
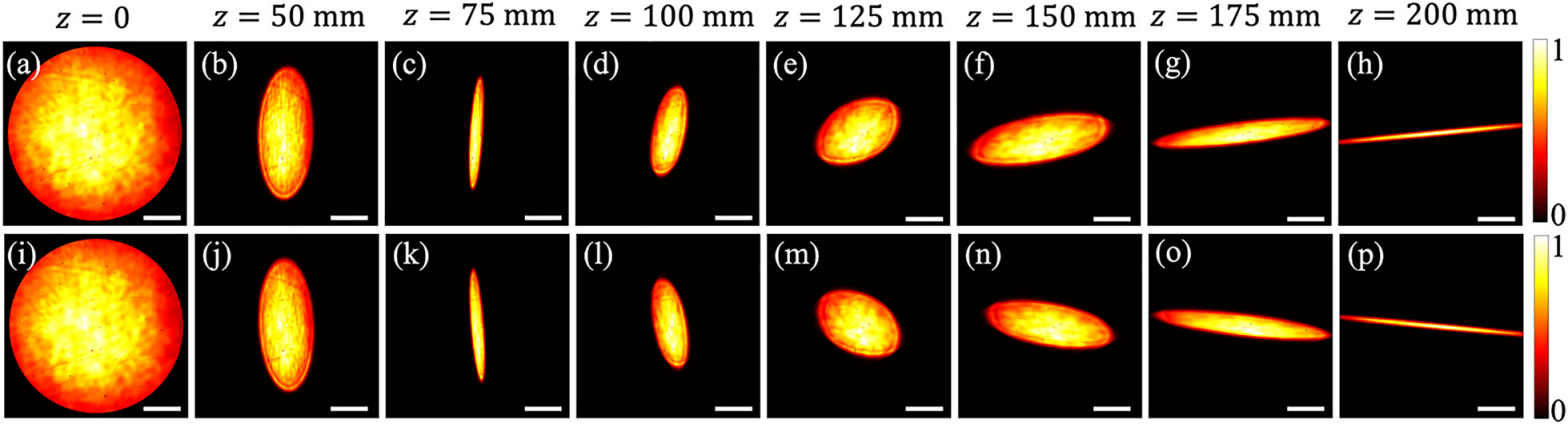
Experimental results of the normalized average intensity distributions of the generated TGSM source with $\mu_{0}=6.25{\text{mm}}^{-2}$ (top) and $\mu_{0}=-6.25{\text{mm}}^{-2}$ (bottom), respectively, at different propagation distances. The scale bars in the figures are 1 mm.

Figure 3(a) and (i) illustrates the experimental results of the intensity distribution of the generated TGSM beams in the source plane with two different twist factors $\mu_{0}=6.25{\text{mm}}^{-2}$ and $\mu_{0}=-6.25{\text{mm}}^{-2}$ , respectively. The other beam parameters set in the CGH are *σ*_0_ = 1.6 mm, *δ*_0_ = 0.4 mm. It is found that the two intensity distributions have the same Gaussian profile that is independent of the value of the twist factor. Nevertheless, the two TGSM beams exhibit different propagation characteristics if the circular symmetry of the beam is broken. In the experiment, we insert a thin lens L_3_ with focal length *f* = 200 mm and a cylindrical lens (CL) with focal length *f*_*x*_ = 150 mm and *f*_*y*_ = ∞, and examine the intensity distributions at different propagation distances after two lenses. The experimental results are shown in Figure 3(a)–(h) and Figure 3(i)–(p). One can see that the rotation directions of the beam spot with respect to the propagation axis are opposite. The positive and negative twist factors correspond to the clockwise and counterclockwise rotating direction, respectively. However, such behavior allows one to judge only the sign of the twist factor. It is difficult to determine the magnitude of the twist factors quantitatively from the propagation intensity distributions.

### 3.2 Measurement of the twist factor

From Eq. (2), it is shown that the twist phase is contained in the phase of the two-point DOC function. To see clearly the role of the twist factor in the DOC, we write Eq. (2) as the more specific form, i.e.,

(10) $$\begin{array}{l} \mu \left(r_{1},r_{2}\right)=\exp\left[-\frac{1}{2\delta_{0}^{2}}{\left|r_{1}-r_{2}\right|}^{2}\right]\\ \times \left\{\cos\left[\mu_{0}\left(x_{1}y_{2}-y_{1}x_{2}\right)\right]-i\sin\left[\mu_{0}\left(x_{1}y_{2}-y_{1}x_{2}\right)\right]\right\}\text{.} \end{array}$$

If we only concentrate on the real part of the DOC and fix the point **r**_2_ = (*x*_2_, *y*_2_) as a reference point, in such a situation, the pattern of the real part looks like an interference pattern of two plane waves with its intensity truncated by a Gaussian profile. The period of the pattern turns out to be

(11) $$T=\frac{2\pi }{\mu_{0}}\sqrt{\frac{1}{y_{2}^{2}}+\frac{1}{x_{2}^{2}}}\text{.}$$

Therefore, by measuring period of the pattern with a known reference point, one could determine the twist factor quantitatively, i.e., $\mu_{0}=\left(2\pi /T\right)\sqrt{x_{2}^{-2}+y_{2}^{-2}}$ .

The real part of the DOC can be measured by interferometry methods. Here, the generated TGSM beam obeys Gaussian statistics. Thus, its DOC can be measured with the help of the famous Hanbury Brown and Twiss (HBT) experiment. The HBT experiment, also known as the intensity correlation between two spatial points, is an efficient way to measure the DOC function of partially coherent light with Gaussian statistics [54], [55], [56]. The relation between the intensity correlation and the DOC is established via Gaussian momentum theorem [54], i.e.,

(12) $$G\left(r_{1},r_{2}\right)=〈I\left(r_{1}\right)I\left(r_{2}\right)〉=S\left(r_{1}\right)S\left(r_{2}\right)+{\left|W\left(r_{1},r_{2}\right)\right|}^{2}\text{,}$$

where S(r)=〈I(r)〉 is the average intensity of the light beam and ${\left|W\left(r_{1},r_{2}\right)\right|}^{2}=S\left(r_{1}\right)S\left(r_{2}\right){\left|\mu \left(r_{1},r_{2}\right)\right|}^{2}$ . It indicates from Eq. (12) that the HBT experiment only measures the modulus of the DOC function. The phase information is lost. In order to recover the phase information or real part of the DOC function, we recently introduced the generalized HBT experiment [52]. In this method, a reference wave is used to interfere with the to-be-detect random field. As shown in part II of Figure 2, a reference beam transmitted from the BS passes the RM_2_, neutral density filter (NDF), and RM_3_, then, coaxially interferes with the generated TGSM beam. A CCD camera records a large number of instantaneous intensity distributions to calculate the intensity correlation function. In this case, the instantaneous mixed electric field can be written as

(13) $$E_{s}\left(r\right)=E\left(r\right)+E_{r}\left(r\right)\text{,}$$

where *E*_*s*_, *E* and *E*_*r*_ represent the mixed field, random field, and reference field, respectively. By applying the Gaussian momentum theorem, the intensity correlation of such mixed field turns out to be (after some mathematical manipulations)

(14) Gs(r1,r2)=〈Is(r1)Is(r2)〉=Ss(r1)Ss(r2)+|W(r1,r2)|2+2Sr(r1)Sr(r2)Re[W(r1,r2)],

where *I*_s_ and *S*_s_ = *S*(**r**) + *S*_*r*_(**r**), respectively, stand for the instantaneous intensity and the average intensity of the mixed field. *S*_*r*_(**r**) is the intensity distribution of the reference field. From Eq. (14), it is found that the last term of the right side contains the real part information of the CSD function. Combining Eqs. (1), (12) and (14), we finally obtain the expression

(15) Re[μ(r1,r2)]=Gs(r1,r2)−G(r1,r2)−S‾(r1,r2)2Sr(r1)Sr(r2)S(r1)S(r2),

where $\bar{S}\left(r_{1},r_{2}\right)=S\left(r_{1}\right)S_{r}\left(r_{2}\right)+S_{r}\left(r_{1}\right)S\left(r_{2}\right)+S_{r}\left(r_{1}\right)S_{r}\left(r_{2}\right)$ . From Eq. (15), one could extract the real part of the DOC function experimentally through the following procedures: First, the average intensity distribution of the reference field *S*_*r*_(**r**) and the random field *S*(**r**) are measured, respectively. Second, the intensity correlations of the random field (the reference arm is closed) and the mixed field are measured, respectively. Finally, the real part of the DOC is evaluated by the measured quantities according to Eq. (15). In the experiment, the CCD records 5000 instantaneous intensity distributions of the random field and the mixed field to calculate the average intensity distribution and the intensity correlation.

**Figure 4:**
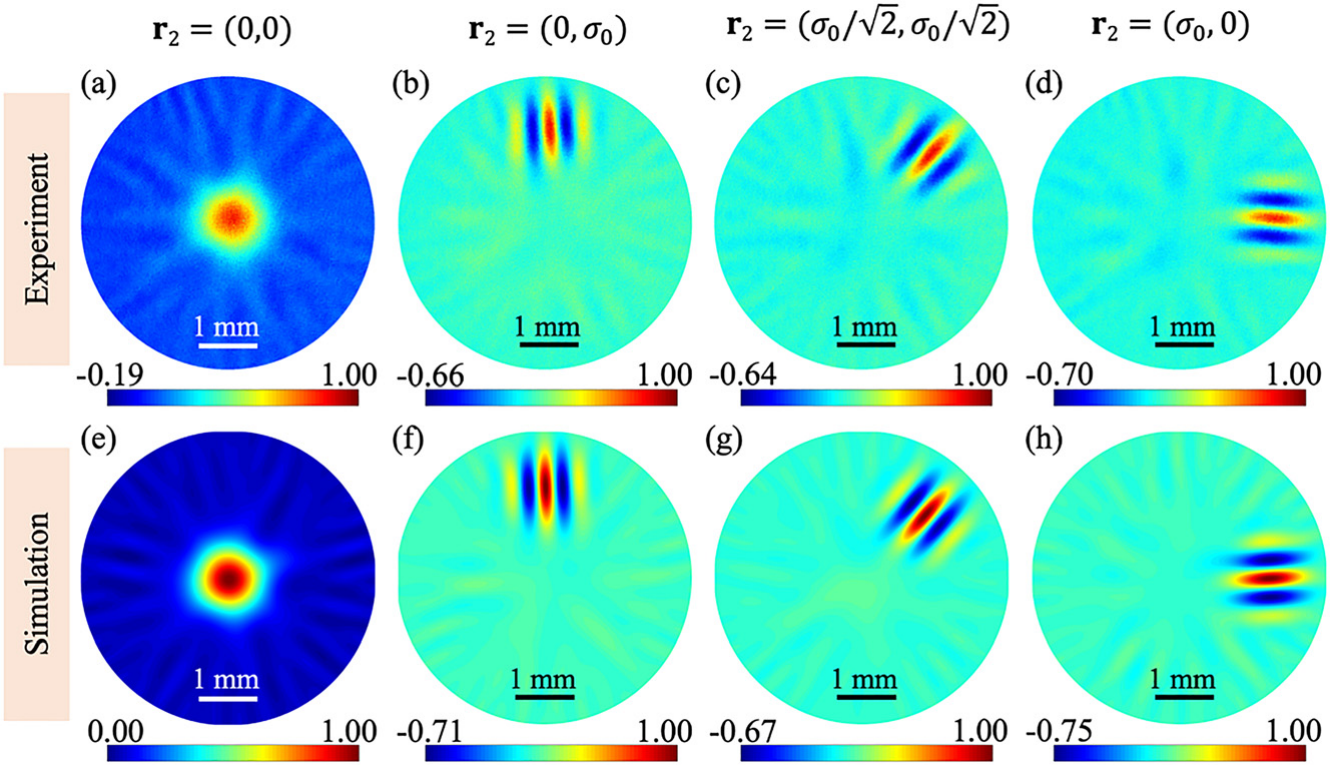
(a)–(d) Experimental and (e)–(h) simulation results of the real part of the DOC of the TGSM beam in the source plane with different reference points $r_{2}$ .

**Figure 5:**
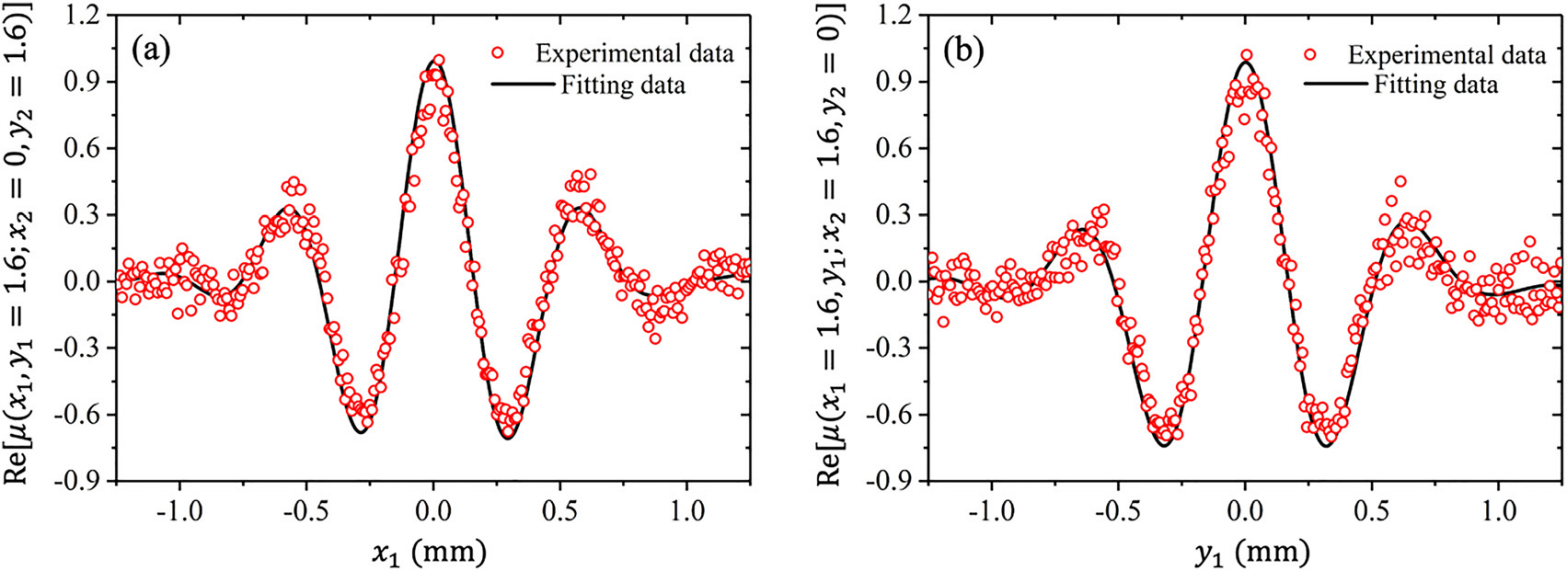
The cross-line of the real part of the DOC function of the TGSM beam when the reference points are selected as (a) $r_{2}=\left(0,\sigma_{0}\right)$ and (b) $r_{2}=\left(\sigma_{0},0\right)$ . The solid curves denote the theoretical fits from the experimental results.

Figure 4(a)–(d) present our experiment results of the real part of the DOC function of the generated TGSM beam in the source plane at four different reference points which are **r**_2_ = (0, 0), (0, *σ*_0_), $\left(\sigma_{0}/\sqrt{2},\sigma_{0}/\sqrt{2}\right)$ , and (*σ*_0_, 0). For convenience of comparison, the corresponding theoretical results are shown in Figure 4(e)–(h). The measured beam width *σ*_0_ is about 1.6 mm. It can be seen from Figure 4 that the real part of the DOC pattern is closely dependent on the specific reference point we choose. When **r**_2_ = (0, 0), the result reduces to a Gaussian profile that is Re[μ(r1,0)]=exp(−r12/2δ02) . The value of beam width *δ*_0_ is obtained through measuring the width of this profile. If the reference point leaves the coordinate origin, the real part of the DOC displays a clear interference pattern, as expected. The line direction of pattern coincides with the radial direction in polar coordinate, whereas the period of the pattern is dependent on the position of reference point as shown in Eq. (11). Note that only a few interference fringes are visible, since the pattern is truncated by a Gaussian function. To determine the twist factor, we plot in Figure 5(a) and (b) the cross-line (red circular dots) of the real part of the DOC in Figure 5(a) at *y* = *σ*_0_ and in Figure 5(b) at *x* = *σ*_0_, respectively. By theoretically fitting the experimental data, we obtain that the twist factor is about 6.33 and 6.12 mm^−2^ in Figure 5(a) and (b), respectively, very closing to the theoretical set value 6.25 mm^−2^. In addition, we also experimentally generate the TGSM beams with different twist factors and measure the twist factors (not shown here). The results show that the measured twist factors agree well with the theoretical setting values, demonstrating the reliability of our generation and measurement method.

It is known from Refs. [35, 57] that the amount of the time-average OAM flux of the TGSM beam along propagation direction is $J_{z}=-2\mu_{0}\;k\sigma_{0}^{2}\;\hbar$ , where k is the wave number. The OAM flux of the TGSM beam is proportional to the twist factor and the square of the beam width. The beam width can be obtained simply from the measurement of the intensity profile of the beam simply. Hence, our method for measuring the twist factor is crucial to determine the OAM flux of the TGSM beam, paving the way to study the transfer of OAM with matter further.

## 4 Conclusions

In summary, we presented an effective way to synthesize the TGSM beam with the help of a single phase-only SLM. The methodology based on the incoherent superposition of random modes obeying Gaussian statistics is discussed. The key in our method is to simultaneously control the amplitude and phase of each random mode (realization) using the phase-only SLM. This allows one to generate the TGSM beams with controllable twist factor without changing the apparatus physically. We validate our method by experimentally generating the TGSM beams with reversed twist phases. The experimental results agree well with the theoretical predictions. Furthermore, we proposed a reliable protocol to quantitively determine the twist factor of the TGSM beam. The kernel of our protocol is to acquire the real part of the DOC from the measurement of the intensity correlations of the mixed fields form by the superposition of a reference wave and the generated random TGSM beam. The twist factor is extracted through evaluating the period of the interference pattern in the real part of the DOC. Our results provide a convenient way to control and detect the second-order phase of a partially coherent light field that are expected to find uses in novel optical imaging based on the coherence phase modulation.
